# The Spectrum of B Cell Functions in Atherosclerotic Cardiovascular Disease

**DOI:** 10.3389/fcvm.2022.864602

**Published:** 2022-04-15

**Authors:** Diede Smeets, Anton Gisterå, Stephen G. Malin, Dimitrios Tsiantoulas

**Affiliations:** ^1^Department of Laboratory Medicine, Medical University of Vienna, Vienna, Austria; ^2^Center for Molecular Medicine, Karolinska University Hospital, Stockholm, Sweden; ^3^Department of Medicine Solna, Karolinska Institutet, Stockholm, Sweden

**Keywords:** B cells, atherosclerosis, antibodies, lipids, inflammation, cardiovascular disease

## Abstract

B cells are a core element of the pathophysiology of atherosclerotic cardiovascular disease (ASCVD). Multiple experimental and epidemiological studies have revealed both protective and deleterious functions of B cells in atherosclerotic plaque formation. The spearhead property of B cells that influences the development of atherosclerosis is their unique ability to produce and secrete high amounts of antigen-specific antibodies that can act at distant sites. Exposure to an atherogenic milieu impacts B cell homeostasis, cell differentiation and antibody production. However, it is not clear whether B cell responses in atherosclerosis are instructed by atherosclerosis-specific antigens (ASA). Dissecting the full spectrum of the B cell properties in atherosclerosis will pave the way for designing innovative therapies against the devastating consequences of ASCVD.

## Introduction

Heart attacks and strokes are the leading causes of mortality and morbidity worldwide (1–3). The main underlying pathology of these clinical manifestations is ASCVD, which leads to the formation of plaques in large and medium-sized arteries. Rupture or erosion of atherosclerotic plaques triggers thrombus formation thereby causing myocardial infarction (MI) or stroke (4, 5). Atherosclerosis is a lipid-driven chronic inflammatory disease characterized by progressive retention of cholesterol-carrying low-density lipoprotein (LDL) particles in the subendothelial space of arteries (6, 7) followed by a chronic maladaptive immune response (4, 8–12) and remodeling of the artery wall (13), fueled by genetic (14) and lifestyle risk factors (13). Local enzymes act on retained lipoproteins, which leads to LDL aggregation and oxidation (OxLDL) characterized by the formation of lipid peroxidation-derived products called oxidation-specific epitopes (OSE) (15, 16). The accumulating modified LDL particles stimulate endothelial cells to produce adhesion molecules and chemokines (17), which attract circulating leukocytes such as T lymphocytes (18) and monocytes (19) to the vessel wall.

The controlled double-blind clinical trials CANTOS (20), COLCOT (21), and LoDoCo2 (22) have demonstrated the therapeutic value of immunomodulation in secondary prevention of ASCVD. While these studies have shown that inflammation is crucially involved in human ASCVD, they also revealed the need for the development of precise immunotherapies that would limit side effects, such as the risk for fatal infections (23).

### B Cells Are Key Pieces of the CVD “Immune-Mosaic”

Patients with autoimmune rheumatic diseases, who display dysregulated responses of adaptive immunity (B and T lymphocytes), are at high risk for premature ischemic heart disease due to accelerated development of atherosclerosis that cannot be fully explained by the traditional Framingham risk factors such as cholesterol levels, smoking and systolic blood pressure (24). Furthermore, mice lacking adaptive immunity display reduced atherosclerosis (25). These findings have highlighted the crucial role of adaptive immunity in modulating atherosclerosis. Several studies have revealed a broad spectrum of T cell [reviewed elsewhere; (18)] and B cell properties that affect atherosclerosis (26–28).

B cells have the unique ability to generate immunoglobulins that can be displayed on the cell surface in the form of the B cell receptor (BCR) or secreted as antibodies. In mouse B lymphopoiesis, B-cell-biased lymphoid progenitors (BLPs) differentiate via the pre-pro-B cell stage to committed pro-B cells, with commitment regulated by the transcription factor *Pax5* (29). The successful display of a recombined heavy chain together with surrogate light chains on the cell surface provides proliferative signals to large pre-B cells and this cell division is followed by rearrangement of the light chain genes at the small pre-B cell stage, hence completing V(D)J recombination and resulting in an immature B cell that displays IgM on the cell surface. Upon completion of recombination events, the B cells can leave the bone marrow to further mature in secondary lymphoid organs. Although the marrow of long bones is often considered the predominant site of B lymphopoiesis, other locations are noticeable for B cell development, including the fetal liver, the calvaria of the skull (30), and also the mouse intestinal lamina propria (31).

Mature B cells consist of two main subsets, the conventional B-2 cells, and the less frequent B-1 cell subset (26). B-1 and B-2 cells display differences in their activation requirements, anatomical localization, and surface markers. B-1 cells are subdivided into B-1a and B-1b cells. B-1a cells are long-lived and self-renewing innate-like B cells that are derived from the fetal liver hematopoiesis, and are enriched within the peritoneal and pleural cavities, although a substantial population also can be found in the spleen (32). Notably, CD20^+^CD27^+^CD43^+^CD70^–^ B cells were proposed to be the equivalent of mouse B-1 cells in humans (33). However, this remains unsettled considering the similarities of CD20^+^CD27^+^CD43^+^CD70^–^ B cells with preplasmablasts (34, 35). On the other hand, B-2 cells display many similarities between mice and humans concerning their localization and function (36). B-2 cells include the follicular (FO) B cells and the marginal zone (MZ) B cells. Both subsets are generated through the maturation of splenic immature B cells, which have successfully escaped the bone marrow selection, via pertinent BCR signaling (37). In contrast to MZ B cells, FO B cells display circulating properties, which allow them to home to distant sites (37).

Early evidence supporting a role for B cells in human atherosclerosis is derived from studies more than 40 years ago that demonstrated the presence of immunoglobulins in atherosclerotic arteries (38, 39). Based on histological analyses, B cells are commonly detected in adventitia surrounding atherosclerotic regions with the ability to recirculate to draining lymph nodes (40), while they are an infrequent cell type in atherosclerotic plaques (41). However, although a technical contamination of circulating B cells cannot be excluded, a mass-cytometry analysis of human carotid atherosclerotic plaques revealed a substantial portion of plaque B cells (42). Besides being present in atherosclerotic arteries, a systems biology investigation of whole blood gene expression data from Framingham Heart Study participants and genome-wide association studies coupled to the construction of co-expression networks, identified coronary heart disease-specific causative modules enriched in genes regulating B-cell activation (43), thereby providing indications for a functional role of B cells in human atherosclerosis. In line with this, numbers of activated CD19^+^CD86^+^ B cells or IgM^+^ unswitched memory B cells display a positive and negative association, respectively, with increased risk for stroke in humans (44, 45), suggesting that B cell activation may be involved in the progression of atherosclerosis.

The first experimental evidence that B cells impact atherosclerosis was provided by Caligiuri et al., who showed that splenectomy-induced acceleration of atherosclerosis in Apolipoprotein E deficient (*Apoe*^–/–^) mice could be rescued upon transfer of splenic B cells that were isolated either from wild type or *Apoe*^–/–^ donors (46). Next, Major et al. reported that lethally irradiated LDL receptor-deficient (*Ldlr*^–/–^) mice that were injected with bone marrow from B cell-deficient (μ*MT*) donor mice developed increased atherosclerosis compared to controls (47). However, in a recent study Tay et al., reported that *Apoe*^–/–^ μ*MT* mice developed decreased atherosclerosis compared to control *Apoe*^–/–^mice (48). *Apoe*^–/–^ mice accumulated predominately VLDL remnants in their circulation whereas in *Ldlr*^–/–^ mice the main accumulating lipoprotein in plasma is the LDL (49). Apart from the obvious reasons, such as different experimental settings, the differences in lipoprotein profile and metabolism may be, at least in part, responsible for the differential effect in atherosclerosis upon B cell deficiency between *Ldlr*^–/–^ and *Apoe*^–/–^mice.

**FIGURE 1:**
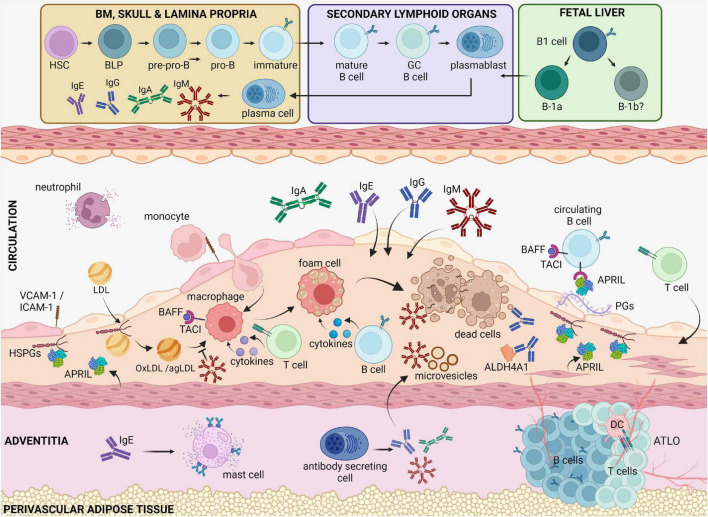
B cell functions in atherosclerotic disease. Circulating cholesterol-containing LDL particles are progressively retained in the subendothelial space of arteries. Oxidized or aggregated LDL particles are taken up by arterial macrophages, which turn to foam cells because of uncontrolled lipid uptake and thereby undergo apoptosis or necrosis. Antibody-producing cells that reside in the bone marrow, the spleen and in the adventitia, produce high amounts of antibodies that are deposited in the plaque. IgM is known to bind and block the proinflammatory effects of oxidized LDL, apoptotic cells and microvesicles. IgE antibodies by binding to FcεRI receptors activate powerful proinflammatory responses by mast cells and macrophages. IgG antibodies can also bind OxLDL as well as self-proteins, such as ALDH4A1, and modulate macrophage activation. BAFF and APRIL, which both bind the TACI receptor in B cells, dampen the proinflammatory responses by macrophages, and limit the LDL retention in the intima, respectively, thereby revealing an indirect property of B cells to regulate plaque inflammation. BLPs, B-cell-biased lymphoid progenitors; PGs, proteoglycans, TACI, Transmembrane activator and CAML interactor; BAFF, B cell activating factor; APRIL, A Proliferation Inducing Ligand, HSPGs, heparan sulfate proteoglycans; BM, bone marrow; ATLO, artery tertiary lymphoid organ; OxLDL, oxidized LDL; agLDL, aggregated LDL.

B cell subsets exhibit distinct effects in atherosclerosis, which further emphasizes the sophisticated involvement of B cells in this disease (Figure 1). B-1 cells confer protection in atherosclerosis (50, 51). On the other hand, treatment of *Apoe*^–/–^ or *Ldlr*^–/–^ mice with a B cell depleting anti-CD20 antibody, which preferentially depletes B-2 cells, reduced atherosclerosis, and prevented the MI-induced acceleration of atherosclerosis (52–54). In addition, genetic deletion of the B cell transcription factor *Pax5* in CD23-expressing cells (primarily mature B2 cells) (55), or treatment with an agonistic antibody specific for B- and T-lymphocyte attenuator (56) that reduced mature B-2 cells, also resulted in decreased atherosclerosis. Disruption of the B cell-activating factor receptor (BAFFR) pathway, which is essential for B-2 (but not B-1) cell survival (57), also conferred an atheroprotective effect (58–61). However, selective ablation of MZ B cells increases atherosclerosis (62), which indicates that therapeutic strategies targeting the entire B-2 cell compartment may not be optimal, and thus, dissecting the functions of B cell responses is essential for the designing of precise therapies in atherosclerosis.

### Antibody-Mediated Functions of B Cells in Atherosclerosis

The main property of B cells that plays a crucial role in atherosclerosis is antibody production. B-1 cells secrete high amounts of natural IgM antibodies, which are produced in absence of a foreign microbial threat (63). On the other hand, FO B cells can enter the germinal center (GC) reaction, which is notable for producing high-affinity antibodies through the process of somatic hypermutation, although this process is not exclusive to the GC. GCs can be found in the secondary lymphoid organs of *Apoe*^–/–^ and *Ldlr*^–/–^ mice (27, 55, 62, 64). Upon exit from the GC reaction, B cells can differentiate into short- and long-lived memory B cells as well as short- and long-lived antibody-producing cells (65).

#### Immunoglobulin M, a Trustworthy Groundkeeper

There is a consensus that IgM exhibits atheroprotective properties. For instance, transfer of B-1a cells into splenectomized mice, which exhibit a severe reduction in peritoneal B-1a cells and circulating IgM antibodies, reversed splenectomy-accelerated atherosclerosis (50). However, the protective effect of B-1a cell transfer in this setting was absent when B-1a cells deficient in secreted IgM (sIgM) were injected (50). Consistent with this, mice lacking sIgM develop aggravated atherosclerosis (66–68). Moreover, B cell-specific CXCR4 (C-X-C chemokine receptor type 4) deficiency, which resulted in reduced IgM levels in plasma, led to increased atherosclerosis in female mice (69). Thus, dissecting the molecular pathways that regulate the production of atheroprotective IgM antibodies may reveal new therapeutic strategies for atherosclerosis. Along this line, apoptotic cell injection (64), infusion of liposomes decorated with phosphatidylserine moieties (70), and genetically induced inhibition of antibody class-switching (71) led to increased total IgM levels in plasma and reduced atherosclerosis. In addition, the transfer of B-1b cells into lymphocyte-deficient atherosclerotic mice also led to increased plasma IgM and reduced plaque size (51). Thus, strategies directly promoting the expansion of B-1a or B-1b cells could be of interest. For instance, reduced atherosclerosis along with increased B-1a cell numbers and circulating IgM levels were reported in atherosclerosis-prone mice that were deficient in sialic acid-binding immunoglobulin-like lectin G (72) or had been treated with an antibody against the phosphatidylserine receptor T-cell immunoglobulin and mucin domain-1 (73). However, therapeutic strategies for the expansion of B-1a cells have to be considered with caution as they may be accompanied by an increase of the proatherogenic B-1a cell-derived subset, the innate response activator (IRA) B cells (74), which via producing granulocyte-macrophage colony-stimulating factor instruct a dendritic cell-mediated promotion of proatherogenic Th1 immunity (75).

The identification of atherosclerosis-specific antigens (ASA) will allow the designing of precise therapeutic strategies in atherosclerosis. Clinical studies have shown an inverse correlation of OSE-specific IgM against malondialdehyde and phosphorylcholine (PC), which are present on oxidized LDL (16, 76) and apoptotic cellular debris (77, 78), with atherosclerotic burden and cardiovascular outcomes (28, 79–82). The implication of OSE-specific IgM antibodies in atherosclerosis was originally shown by using the E06 IgM antibody that binds oxidized phospholipids (OxPLs) and has an identical CDR3 region to the germline-encoded B-1 cell-derived T15 clone (83). Immunization with heat-killed pneumococcal extracts led to a strong increase of the PC-specific T15/E06 IgM clonotype and decreased lesion formation (84). Furthermore, passive infusion of T15/E06 IgM antibodies reduced vein graft atherosclerosis in atherosclerotic *Apoe*^–/–^ mice thereby providing direct evidence that the E06 IgM confers an atheroprotective effect (85). In a seminal study by Prof. Witztum’s lab, it has been shown that transgenic overexpression of the single-chain variable fragment of E06 strongly decreased atherosclerosis in *Ldlr*^–/–^ mice (86). These data suggest that E06 acts as a blocking antibody limiting the proinflammatory effect of OxPLs in atherosclerosis *in vivo*. This is supported by the capacity of E06 to block OxLDL uptake by macrophages (87) and proinflammatory cytokine production by OxPL-stimulated macrophages (88) *in vitro.* While the expansion of OSE-specific IgM could be considered therapeutically in atherosclerosis, it is important to identify its right “therapeutic window.” This is essential as endogenous OSE-specific IgMs are present at high levels in both mice and humans (89) and increase over time in hypercholesterolemia (90). In fact, infusion of purified T15/E06 preparations (91) or the OxPL-neutralizing 10C12 IgM clone (68) had no effect in advanced atherosclerosis. Furthermore, it appears likely that long exposure to an atherogenic milieu might induce the expansion of IgM with shared antigen specificities. For instance, genetic deficiency of the *V_*H*_S107.1.42* locus, which is essential for the successful production of T15 antibodies, did not affect experimental atherosclerosis in mice with marked dyslipidemia (92). IgMs also recognize other self-antigens and thereby regulate the maturation of B-2 cells (93–96) and circulating levels of other immunoglobulins (67, 97, 98). Notably, mice lacking sIgM, display high levels of IgE antibodies, which are responsible for the accelerated atherosclerosis in this setting (67). Taken together, the properties of IgM in atherosclerosis demonstrate the important role of (neo)-self-antigens in this disease.

#### Immunoglobulin E, a Powerful Assailant

The most well-documented properties of IgE antibodies are their role in triggering an allergic reaction and fighting microbial infections (99, 100). These properties of IgE are mediated via binding to high-affinity IgE receptor FcεRI, which is mainly present on mast cells, basophils, and eosinophils (101). Mice lacking the FcεRI receptor display reduced atherosclerotic plaque size, thereby suggesting that IgE antibodies play a role in atherosclerosis (102). The proatherogenic role of IgE antibodies was directly shown using a neutralizing anti-IgE antibody specific for free IgE that, as mentioned above, completely reversed the accelerated atherosclerosis in atherosclerotic sIgM deficient mice, which display high plasma IgE antibodies (67). In agreement with this, mice deficient in IgE antibodies developed decreased atherosclerosis (103). Furthermore, a systemic IgE-mediated mast cell activation in atherosclerotic mice lacking B cells resulted in increased lesion size (104). Mechanistically, IgEs promote mast cell and neutrophil activation, and the production of proinflammatory cytokines by macrophages and smooth muscle cells (67, 102, 103), which could be responsible for their effect in atherosclerosis *in vivo*. The detrimental role of IgE antibodies in atherosclerosis is also supported by several epidemiological studies (105, 106) that also implicate the mammalian oligosaccharide galactose-α-1,3-galactose as a candidate antigen (107). Future studies are required to identify the spectrum of proatherogenic IgE-specific antigens.

#### Immunoglobulin G, a Vault for Atherosclerosis-Specific Antigens?

IgG antibodies are produced in different subclasses: IgG1, IgG2, IgG3, and IgG4 in humans and IgG1, IgG2a/c, IgG2b, and IgG3 in mice (108). Tay et al., provided the first direct evidence on the role of IgG antibodies in atherosclerosis, by showing that mice lacking most endogenous immunoglobulins developed increased plaque size upon injection of purified total IgG from atherosclerotic mice compared to IgG from non-atherosclerotic donors (109). This study also suggests that exposure to an atherosclerotic milieu alters the antigen specificities of the IgG repertoire by inducing the expansion or even the *de novo* generation of B cell clonotypes that are likely to include specificities against ASA. In line with this, a protein array analysis revealed an altered repertoire of IgG1 protein targets in the serum of *Apoe*^–/–^ vs. C57BL/6 mice fed an atherogenic diet (71). It is not clear whether the altered IgG repertoire is triggered upon chronic exposure to atherogenic pressure or already emerges at the initiation of the disease. In this regard, abrupt loss of APOE, which results in acute onset of dyslipidemia, triggered a rapid increase in IgG antibodies levels enriched in specificities against common autoantigens (55). These data suggest that the GC reaction is involved in atherosclerosis. In agreement with this, elimination of GC B cells achieved upon deletion of the key B cell transcription factor *Pax5* in AID-expressing B cells, reduced atherosclerosis (55, 110). Furthermore, B cell-specific overexpression of the FcγRIIB receptor limited GC B cell responses and reduced atherosclerosis in male mice (111). Moreover, deletion of *Prdm1* encoding the key transcription factor BLIMP1, in all B cells (110) or selectively in mature B cells (109), caused impaired plasma cell differentiation and a dramatic reduction in all immunoglobulin isotypes (particularly in IgG) and led to reduced atherosclerotic plaque size (109, 110). While these data show that the IgG antibodies confer an overall proatherogenic effect, B cell-specific deletion of the transcription factor x-box binding protein-1, which similarly to BLIMP-1 deficiency resulted in reduced levels of all immunoglobulins, increased early atherosclerosis (112). Thus, it is conceivable that the IgG repertoire also includes atheroprotective clones. Indeed, Lorenzo et al. have recently shown that GC-derived antibodies from hypercholesterolemic mice against mitochondrial dehydrogenase ALDH4A1 protect from atherosclerosis (27), thereby demonstrating that the repertoire of the antigen specificities of GC B cells includes also protective responses in atherosclerosis. This conclusion is also supported by Centa et al. showing that IgG antibodies could promote plaque stability (110). It appears promising that the identification of ASA recognized by IgG would reveal new mechanistic layers for the role of B cell responses in atherosclerosis.

### Antibody-Independent Functions of B Cells in Atherosclerosis

B cells are an important source of cytokines (113). Transfer of CD21^hi^CD23^hi^CD24^hi^ IL-10 secreting B cells isolated from renal lymph nodes into syngeneic mice increased plaque size in a perivascular collar injury model of the carotid artery (114). In contrast, B cell-specific IL-10 deficiency did not affect atherosclerosis in the aortic root (115). These data suggest that B cells may exhibit distinct effects in different atherosclerosis-prone sites. B-2 cell functions that affect atherosclerosis, such as antigen presentation via MHCII complexes (48, 109), CD40 (48, 109), and GITRL (116) signaling, involve interaction with T cells. In addition, MZ B cells mediate their protective effect in atherosclerosis via suppressing the proatherogenic responses of T follicular helper cells (62). However, lymphocyte-deficient mice that were injected with splenic B-2 cells developed increased atherosclerosis (53), which shows that B-2 cells can affect plaque formation in absence of T cells, for example via the production of TNF (117). Furthermore, BAFFR deficiency or blockage (that leads to dramatically reduced B cell numbers) limits atherosclerosis (58–60), whereas soluble BAFF neutralization aggravates atherosclerotic plaque size (118). Both B cell deficiency (48) as well BAFFR blockage (60) result in increased soluble BAFF levels that could be responsible for the atheroprotective effect in these settings. Similarly, A Proliferation Inducing Ligand (APRIL), which is recognized by B cells through shared receptors with BAFF, confers atheroprotection via binding to heparan-sulfate proteoglycans (HSPGs) in the artery wall (119). Therefore, it is likely that B cell depletion in the vessel wall (120, 121) would increase the availability of APRIL for binding to HSPGs.

### Summary and Future Perspectives

B cells have the capacity to sense and respond to atherosclerosis. The revolutionary development of high-throughput technologies and methods for gene editing will allow the dissection of the mechanisms by which B cells impact atherosclerotic plaque formation in an unprecedented depth. A key point in this effort would be to identify if and how the B cell response in atherosclerosis is driven by ASA. This will set the ground for the development of precise therapies that will target selectively culprit or atheroprotective B cell clones.

## Author Contributions

DS, AG, SM, and DT researched the data, wrote, and reviewed the manuscript. DS designed the figure (created with BioRender.com) with input from the other authors. DT supervised the preparation of the manuscript. All authors contributed to the article and approved the submitted version.

## Conflict of Interest

DT was a named inventor on a patent application (EP20217536.0) to exploit APRIL for diagnostic and therapeutic purposes in cardiovascular diseases that has been filed by the Medical University of Vienna (Austria) and CeMM Research Center for Molecular Medicine of the Austrian Academy of Sciences (Austria). The remaining authors declare that the research was conducted in the absence of any commercial or financial relationships that could be construed as a potential conflict of interest.

## Publisher’s Note

All claims expressed in this article are solely those of the authors and do not necessarily represent those of their affiliated organizations, or those of the publisher, the editors and the reviewers. Any product that may be evaluated in this article, or claim that may be made by its manufacturer, is not guaranteed or endorsed by the publisher.
